# Structure Elucidation and Functional Studies of a Novel β-hairpin Antimicrobial Peptide from the Marine Polychaeta *Capitella teleta*

**DOI:** 10.3390/md18120620

**Published:** 2020-12-04

**Authors:** Pavel V. Panteleev, Andrey V. Tsarev, Victoria N. Safronova, Olesia V. Reznikova, Ilia A. Bolosov, Sergei V. Sychev, Zakhar O. Shenkarev, Tatiana V. Ovchinnikova

**Affiliations:** 1 M.M. Shemyakin & Yu.A. Ovchinnikov Institute of Bioorganic Chemistry, the Russian Academy of Sciences, Miklukho-Maklaya str., 16/10, 117997 Moscow, Russia; ibch@inbox.ru (P.V.P.); tsarev@nmr.ru (A.V.T.); victoria.saf@ibch.ru (V.N.S.); olesya-r@nmr.ru (O.V.R.); bolosov@ibch.ru (I.A.B.); svs@ibch.ru (S.V.S.); zakhar.shenkarev@nmr.ru (Z.O.S.); 2 Department of Biotechnology, I.M. Sechenov First Moscow State Medical University, Trubetskaya str., 8–2, 119991 Moscow, Russia

**Keywords:** antimicrobial peptide, polychaeta, innate immunity, BRICHOS domain, recombinant peptide, β-hairpin structure, nuclear magnetic resonance (NMR)

## Abstract

Endogenous antimicrobial peptides (AMPs) are evolutionary ancient molecular factors of innate immunity that play a key role in host defense. Among the most active and stable under physiological conditions AMPs are the peptides of animal origin that adopt a β-hairpin conformation stabilized by disulfide bridges. In this study, a novel BRICHOS-domain related AMP from the marine polychaeta *Capitella teleta*, named capitellacin, was produced as the recombinant analogue and investigated. The mature capitellacin exhibits high homology with the known β-hairpin AMP family—tachyplesins and polyphemusins from the horseshoe crabs. The β-hairpin structure of the recombinant capitellacin was proved by CD and NMR spectroscopy. In aqueous solution the peptide exists as monomeric right-handed twisted β-hairpin and its structure does not reveal significant amphipathicity. Moreover, the peptide retains this conformation in membrane environment and incorporates into lipid bilayer. Capitellacin exhibits a strong antimicrobial activity in vitro against a wide panel of bacteria including extensively drug-resistant strains. In contrast to other known β-hairpin AMPs, this peptide acts apparently via non-lytic mechanism at concentrations inhibiting bacterial growth. The molecular mechanism of the peptide antimicrobial action does not seem to be related to the inhibition of bacterial translation therefore other molecular targets may be assumed. The reduced cytotoxicity against human cells and high antibacterial cell selectivity as compared to tachyplesin-1 make it an attractive candidate compound for an anti-infective drug design.

## 1. Introduction

The spike in antimicrobial resistance along with challenges of conventional antibiotics discovery point out the urgent need for development of new approaches to control infections. Among them, implementation of AMPs which are important components of the innate host defense in many organisms including humans [1]. Invertebrates lack acquired immunity and rely on their innate immunity on pathogens invasion, and AMPs play a key role in such a rapid immune response. In contrast to other marine invertebrate animals, polychaeta is a relatively underexplored class in terms of discovery of new host-defense peptides. AMPs have been identified in several species of polychaetes: 21-residue β-hairpin arenicins from *Arenicola marina* [2], 22-residue β-hairpin alvinellacin from *Alvinella pompejana* [3], 33-residue nicomicins from *Nicomache minor*, combining an amphipathic *N*-terminal α-helix and *C*-terminal extended part with a six-residue loop stabilized by a disulfide bridge [4], 51-residue perinerin from *Perinereis aibuhitensis*, 22-residue α-helical hedistin containing bromo-tryptophans from *Nereis diversicolor* [5], and theromacin from *Perinereis linea*—a first representative of the cysteine-rich macin family among polychaeta species [6].

Here we described a novel β-hairpin antimicrobial peptide, named capitellacin, that was earlier predicted based on the genome data of the polychaeta *Capitella teleta* [3]. *C. teleta* is a small segmented worm found in the sediment of organically enriched areas such as sewage treatment plants, and is well known as a bioindicator of disturbed habitats [7]. This worm is also used as a model organism in tissue regeneration studies [8]. Capitellacin exhibits the highest homology with tachyplesins and polyphemusins–known β-hairpin AMP families, both originating from the horseshoe crabs [9]. At the same time, like arenicins, alvinellacin, and nicomicins, capitellacin is synthesized as the *C*-terminal part of corresponding precursor protein containing the BRICHOS domain [4]. Therefore, all the above-mentioned peptides could be designated as BRICHOS domain-related antimicrobial peptides. The precursor proteins that contain the BRICHOS domain are characterized by low sequence identities but highly conserved overall domain architecture [10]. BRICHOS domain presumably functions as intramolecular chaperone guiding the aggregation-prone region, in particular a hydrophobic AMP part, into the correct conformation during biosynthesis.

In this study, the most probable variant of the natural mature capitellacin processed by furin protease was expressed as the recombinant peptide in *Escherichia coli*. The peptide structure in aqueous solution and membrane environment was investigated by NMR, CD, FTIR, and fluorescent spectroscopy. A comparative analysis of biological activity of capitellacin and tachyplesin-1 was performed. In particular, antibacterial activity against a wide panel of ESKAPE pathogens as well as cytotoxic properties of capitellacin were analyzed. We also compared the ability of the peptides to compromise the integrity of bacterial membranes using both living cells and proteoliposome model. The effect of capitellacin on bacterial translation was accessed using the cell-free enhanced green fluorescent protein (EGFP) expression system.

## 2. Results and Discussion

### 2.1. Recombinant Production of the Capitellacin

**Figure 1:**
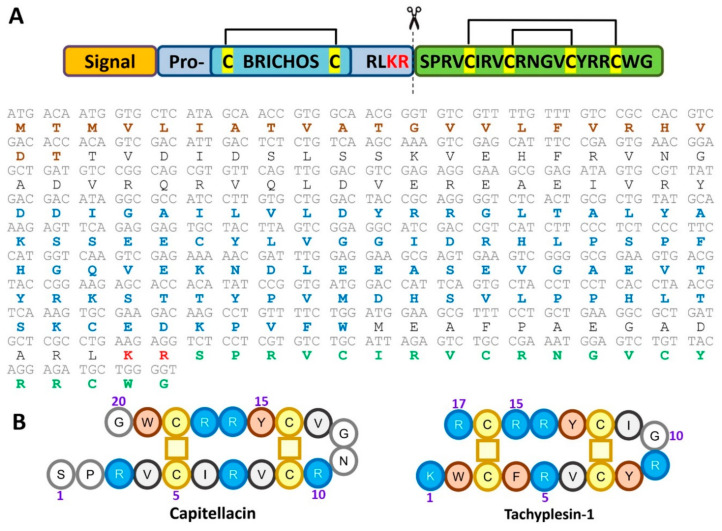
(**A**) Capitellacin precursor protein organization and amino acid sequence. The signal peptide sequence identified with SignalP-5.0 (http://www.cbs.dtu.dk/services/SignalP/) and the BRICHOS domain sequence identified with MyHits Motif Scan (https://myhits.isb-sib.ch/cgi-bin/motif_scan) are highlighted with brown and blue, respectively. The putative natural processing site is indicated with scissors. (**B**) Primary structure of capitellacin in comparison with that of tachyplesin-1.

Earlier the nucleotide sequence encoding capitellacin was identified by blasting the preproalvinellacin in the *C. teleta* whole genome shotgun (WGS) database [3]. Expression of the preprocapitellacin gene was verified by analysis of *C. teleta* transcripts (NCBI sequence read archives SRX2653646-SRX2653653) deposed in GenBank with the use of nucleotide BLAST instruments. The nucleotide sequence of procapitellacin corresponded to data obtained by genome sequencing (GenBank: AMQN01012506.1, Figure 1A). The sequence analysis with the use of SignalP 5.0 pointed to the Thr22-Thr23 bond as the most probable cleavage site for eukaryotic signal peptidase. In its turn, procapitellacin could be processed by furin protease that frequently cleaves the peptide bond after the dibasic Lys-Arg site. Thus, the 20-residue *C*-terminal fragment of procapitellacin was chosen for further investigations as the most probable variant of the natural mature peptide. Bioinformatic analysis revealed that the mature capitellacin does not bear similarity higher than ~52% with any known AMPs listed in different AMP databases. The peptide exhibits the highest homology with tachyplesin-1 from the horseshoe crab *Tachypleus tridentatus* (Figure 1B).

Like other known polychaeta β-hairpin AMPs, in particular arenicin-3 and alvinellacin, the peptide is stabilized by two disulfide bonds [11]. In this study, we used bacterial heterologous expression of target peptides fused with modified thioredoxin A–a highly soluble carrier protein that was successfully used for recombinant production of other β-hairpin AMPs [12]. The fusion proteins were expressed in *E. coli* BL21 (DE3) cells, and the obtained total cell lysates were fractionated by affinity chromatography. After purification and cleavage of the fusion proteins, reverse-phase high performance liquid chromatography (RP-HPLC) was used to obtain target AMPs. MALDI mass spectrometry analysis of the main fractions showed that the measured monoisotopic m/z matched well with the calculated molecular masses of protonated ions [M + H]^+^ of corresponding peptides indicating formation of two disulfide bonds and the absence of any other modifications (Table 1, Figure S1). The final yield of the capitellacin was of 6.1 mg per 1 L of the culture that is comparable to that of tachyplesin-1.

### 2.2. Spatial Structure of the Capitellacin in Aqueous Solution

**Figure 2:**
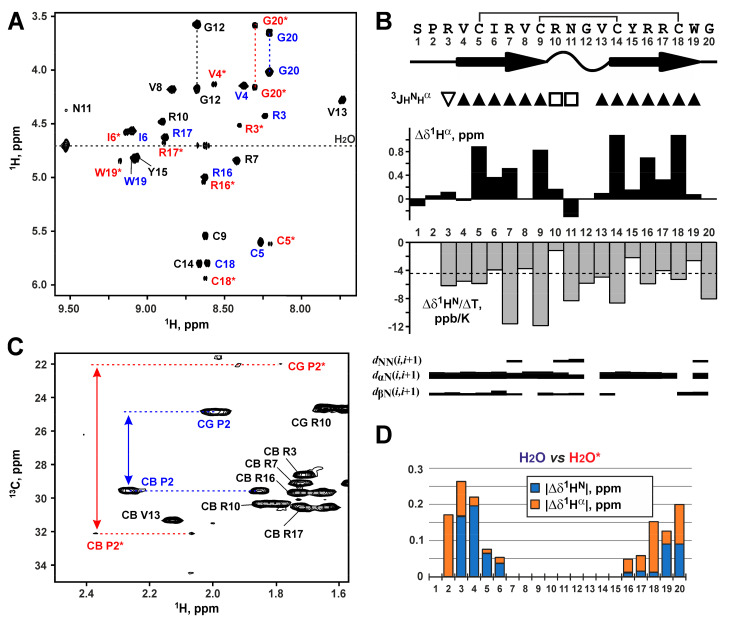
NMR data define the secondary structure and conformational heterogeneity of capitellacin in solution. (**A**). The fragment of the 2D ^1^H-TOCSY spectrum of 1.5 mM capitellacin (5% D_2_O, pH 5.2, 30 °C). The resonance assignment of H^N^-H^α^ cross-peaks is shown. The signals of the major and minor forms of the peptide are marked in blue and red, respectively. The signals of the minor form are additionally marked with asterisks. The signals common to both forms are marked in black. (**B**). Overview of obtained NMR data. (From top to bottom) Secondary structure of capitellacin. The β-sheets are denoted by arrows and tight β-turn by wavy line. (^3^J_H_^N^_H_^α^) Large (>8 Hz), small (<6 Hz), and medium (others) coupling constants are indicated by the filled triangles, open triangles, and open squares, respectively. (Δδ^1^H^α^) Secondary chemical shifts of ^1^H^α^ nuclei. The positive values revealed formation of β-structure. (∆δ^1^H^N^/∆T) Temperature gradients of amide protons. The values with amplitude <4.5 ppb/K (dashed line) indicated possible participation of HN group in the hydrogen bond formation. The NOE connectivities are shown as usual. The width of the lines corresponds to the relative intensity of the cross-peak in the 150 ms NOESY spectrum. **(C)**. The fragment of the 2D ^13^C-HSQC spectrum of capitellacin at natural isotope abundance. The resonance assignment of the signals of H_2_C^β^ and H_2_C^γ^ groups of Pro2 residue for the major and minor form of the peptide are marked in blue and red, respectively. The difference of chemical shifts between ^13^C^β^ and ^13^C^γ^ nuclei in both forms are shown. (**D**). Difference (absolute value) of ^1^H^N^ (blue) and ^1^H^α^ (orange) chemical shifts between the two forms of capitellacin in solution.

Analysis of the NMR spectra of capitellacin in water revealed the presence of two structural forms of the peptide (Figure 2A). The relative population of the two forms was about 1:5. Complete ^1^H and partial ^13^C resonance assignments of both peptide forms were obtained at pH 5.2 and 30 °C. A summary of the obtained NMR data is shown in Figure 2B. The ^13^C^β^ resonances of Cys5, Cys9, Cys14, and Cys18 residues of the major form and ^13^C^β^ resonances of Cys5 and Cys18 residues of the minor form were observed in the chemical shifts range from 47 to 49 ppm, which are characteristic for the oxidized Cys residues forming disulfide bonds. The chemical shifts of Cys9 and Cys14 residues in both forms were identical.

Large positive values of the secondary chemical shifts of ^1^H^α^ nuclei, characteristic pattern of medium- and long-range nuclear Overhauser effect (NOE) contacts, and the values of the ^3^J_H_^N^_H_^α^ coupling constants exceeding 8 Hz (Figure 2B) suggested the formation of an antiparallel β-structure in the major peptide form. The antiparallel arrangement is in agreement only with the laddered (Cys5-Cys18 and Cys9-Cys14) disulfide bond pattern. Thus, the major form of capitellacin in solution has the β-hairpin conformation with Arg10-Val13 residues on its tip. The observed values of temperature coefficients of amide protons (∆δ^1^H^N^/∆T) revealed that HN groups of Ile6, Val8, Arg10, Tyr15, Arg17, and Trp19 residues could participate in the hydrogen bond formation. This pattern also agrees well with the β-hairpin conformation.

Comparison of the ^1^H^N^ and ^1^H^α^ chemical shifts between both forms of capitellacin in solution showed that the structural differences between the forms are greater at the *N*- and *C*-termini of the peptide and decrease towards the tip of the β-hairpin (Figure 2D). Analysis of the chemical shifts of ^13^C^β^ and ^13^C^γ^ nuclei of the Pro2 residue (Figure 2C) revealed that Ser1-Pro2 peptide bond in the major form has a *trans*- configuration (δ^13^C^β^ − δ^13^C^γ^ = 4.7 ppm), while this bond in the minor form has *cis*- configuration (δ^13^C^β^ − δ^13^C^γ^ = 10.1 ppm). Thus, observed conformational heterogeneity of capitellacin in solution is the consequence of *cis-trans* isomerization of Ser1-Pro2 peptide bond. The characteristic time of corresponding exchange process exceeds 200 ms.

**Figure 3:**
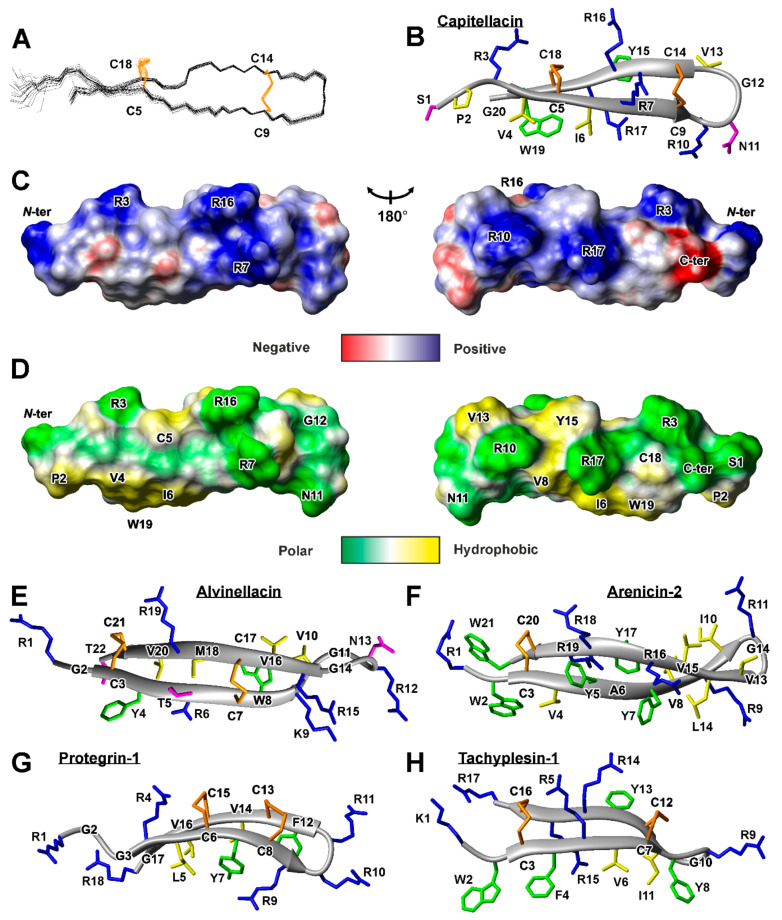
Spatial structure of capitellacin in comparison with other animal β-hairpin antimicrobial peptides. (**A**). The set of the best 20 capitellacin structures superimposed over the backbone atoms. Disulfide bonds are shown in orange. (**B**). The representative conformer of capitellacin in ribbon representation. The disulfide bonds, positively charged, hydrophobic, aromatic, and polar residues are colored in orange, blue, yellow, green, and magenta, respectively. (**C**,**D**) Two-sided view of molecular surface of capitellacin. Electrostatic **(C)** and molecular hydrophobicity (**D**) potentials are shown [13]. Red, blue, green, and yellow areas denote negative, positive, polar, and hydrophobic regions, respectively. Spatial structure of β-hairpin antimicrobial peptides alvinellacin (**E**), arenicin-2 (**F**), protegrin-1 (**G**), and tachyplesin-1 (**H**). PDB codes 7ALD, 2LLR, 2JNI, 1PG1, and 2RTV for capitellacin, alvinellacin, arenicin-2, protegrin-1, and tachyplesin-1, respectively.

The set of 20 capitellacin structures was calculated in the CYANA from 200 random starts using upper NOE-based distance restraints, torsion angle restraints, hydrogen and disulfide bond restraints (Figure 3A and Table S1). The peptide represents β-hairpin, formed by two β-strands (Val4-Cys9 and Cys14-Trp19). The tip of the β-hairpin (Arg10-Val13) is folded in type IV β-turn (Figure 2B). The disulfide bridges (Cys5-Cys18 and Cys9-Cys14) join residues opposite one another on the antiparallel β-strands and have a “short right-handed hook” or g+npng+ conformation (see the Material and Methods for the definition). The capitellacin structure is additionally stabilized by six backbone-backbone hydrogen bonds. The structure is well defined in the region enclosed by Cys5-Cys18 disulfide, while *N*- and *C*-terminal fragments show some disorder (Figure 3A).

The two-stranded β-sheet in the capitellacin structure has a pronounced right-handed twist (Figure 3A,B). The primary structure of the peptide basically contains two types of amino acid residues: hydrophobic/aromatic and positively charged. In spite of this, analysis of the surface properties (Figure 3C,D) did not reveal significant amphipathicity of the capitellacin molecule in aqueous solution. The charged moieties have an almost uniform distribution on the peptide surface (Figure 3C), leaving only one hydrophobic cluster formed by the side-chains of Pro2, Val4, Ile6, Val8, and Trp18 (Figure 3D).

Several AMPs from annelids having β-hairpin structure were previously studied by NMR spectroscopy in aqueous solution. The structures of arenicin-2 from *A. marina* and alvinellacin from *A. pompejana* are shown in the Figure 3E,F. Similar to capitellacin, both peptides contain disulfide-stabilized two-stranded antiparallel β-sheet. Interestingly, all the three peptides demonstrate right-handed twist of the β-structure. The arenicin-2 molecule has the largest twist of 213 ± 8° (per 8 residues, ~27°/res) [14], while the twist angles in the alvinellacin and capitellacin structures are smaller 98 ± 34° (per 6 residues, ~17°/res) and 127 ± 10° (per 7 residues, ~18°/res), respectively. This can be connected with larger degree of β-structure stabilization provided by two disulfides present in the alvinellacin and capitellacin molecules, as compared with only one disulfide in the arenicin-2 molecule (Figure 3B,E,F). In line with this, other β-hairpin AMPs with two disulfide bridges, protegrin-1 and tachyplesin-1 (Figure 3G,H), also demonstrate relatively small right-handed twist ~14° and ~7° per residue, respectively.

Presently, it is generally accepted that a cellular membrane is the main target of the β-hairpin AMPs [11]. Previous NMR studies of arenicin-2 [15] and porcine protegrin-1 [16] revealed that β-hairpin AMPs can form dimers in the membrane environment which combines into the larger ion-conducting oligomeric pores. Significant untwisting of the arenicin-2 β-sheet was observed upon the dimer formation in the membrane mimicking media of detergent micelles; this results in the almost flat β-structural dimers [15]. Despite differences in the dimer structures (CN↑↑NC type of association for arenicin-2 and NC↑↑CN type of association for protegrin-1) in both cases β-strands predominantly composed of hydrophobic residues (and not containing charged or polar ones) are involved in the dimerization interface. Notably, the capitellacin sequence lacks such structural elements. The longest hydrophobic fragments (Val4-Ile6 and Val13-Tyr15) are composed of three residues which is insufficient for dimerization. Thus, it can be assumed that capitellacin dimerization is unlikely upon interaction with cell membranes. Indeed, for a number of β-hairpin peptides, it has been shown that the ability to form dimers is not a necessary condition for both membranolytic action and high antibacterial activity. Recently, we obtained the arenicin-1 analogue Val8Arg which had significantly lower dimerization propensity and cytotoxicity against mammalian cells [17]. At the same time, this analogue exhibited similar antibacterial activities and kinetics of bacterial membrane permeabilization as compared with wild-type arenicin.

### 2.3. Capitellacin Interaction with Model Membranes

**Figure 4:**
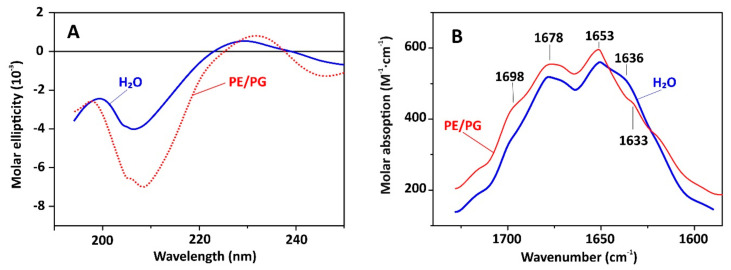
CD spectra (**A**) and FTIR spectra in amide I region (**B**) of capitellacin in water and in soybean PE/PG (2:1) liposomes.

To characterize capitellacin interaction with lipid bilayers we used several technics: circular dichroism (CD), FTIR, and Trp fluorescence spectroscopy. In this study, PE/PG (2:1) lipids mimicking plasma membrane of *E. coli* were utilized. As presented in Figure 4A, the CD spectra of capitellacin dissolved in water showed a negative peak at 207 nm and a positive one at 230 nm. It is known that the backbone structure has a basic influence on the n→π٭ and π→π٭ rotational strengths. The negative CD band at ~210 nm is typical for β-turns [18]. The addition of PE/PG lipids resulted in a significant increase in the intensity that indicates the formation of more rigid structure in lipid bilayer (Figure 4A). The same spectrum overall shape with negative band at 205 nm and positive band at 230 nm was earlier shown for another structurally related β-hairpin AMP gomesin from the spider *Acanthoscurria gomesiana* [19]. Notably, the increase in the intensity was also observed in the presence of POPG or POPC lipids [19]. Gomesin is known as non-dimerizing 18-residue peptide with highly rigid structure stabilized with two disulfide bridges and the flexible Arg-Gly-Arg *C*-termini [20].

While the CD spectra of capitellacin point only to formation of β-structure, the FTIR spectra provide more precise information. The spectra measured in amide I (C = O stretch) region (Figure 4B) have three peaks: band at 1630–1636 cm^−1^ and ~1650 cm^−1^ corresponds to the absorbance of the backbone CO groups in the β-structure, and another one at 1670–1678 cm^−1^ is attributed to free amide carbonyls which are not involved in intramolecular H-bonds [21,22]. Similarity of the FTIR spectra of the peptide in water and in PE/PG membrane indicates stability of its structure when passing into the lipid bilayer. This is in a sharp contrast to arenicins, dimerization of which in membrane-mimicking environments was shown to lead to significant changes in the appearance of FTIR spectra [21,22].

Localization of capitellacin in PE/PG (2:1) lipid bilayer was studied by Trp fluorescence (Table 2). The peptide contains only one Trp19 residue located in the *C*-terminal part of the molecule. It is well known that change of polarity of the indole ring microenvironment led to blue shift from λ_max_ ~350 nm in an aqueous solution to λ_max_ ~330 nm in a hydrophobic environment. Intermediate position of the emission peak at 335–340 nm corresponds to an interface localization of Trp side chain or intermediate polarity of the solvent. In line with it, the λ_max_ values of 350 and 335 nm were observed for capitellacin in water and methanol (Table 2). The value of 332 nm observed in PE/PG bilayer indicates that the Trp residue of capitellacin is located in the hydrophobic acyl chain region of the membrane. Stern-Volmer constants (K_sv_) of the Trp fluorescence quenching by iodide ions which do not penetrate into the hydrophobic region of bilayer are shown in Table 2. The K_sv_ value obtained for capitellacin in water (9.9 M^−1^) was slightly higher than K_sv_ for isolated Trp in water (9.1 M^−1^). Probably the quenching is enhanced by positive charges of the neighboring arginine residues. More than 3-times decrease of K_sv_ upon the peptide transfer from water to the lipid membrane confirms incorporation of capitellacin into PE/PG bilayer.

### 2.4. Conformation of Capitellacin in Membrane-mimicking Detergent Micelles

**Figure 5:**
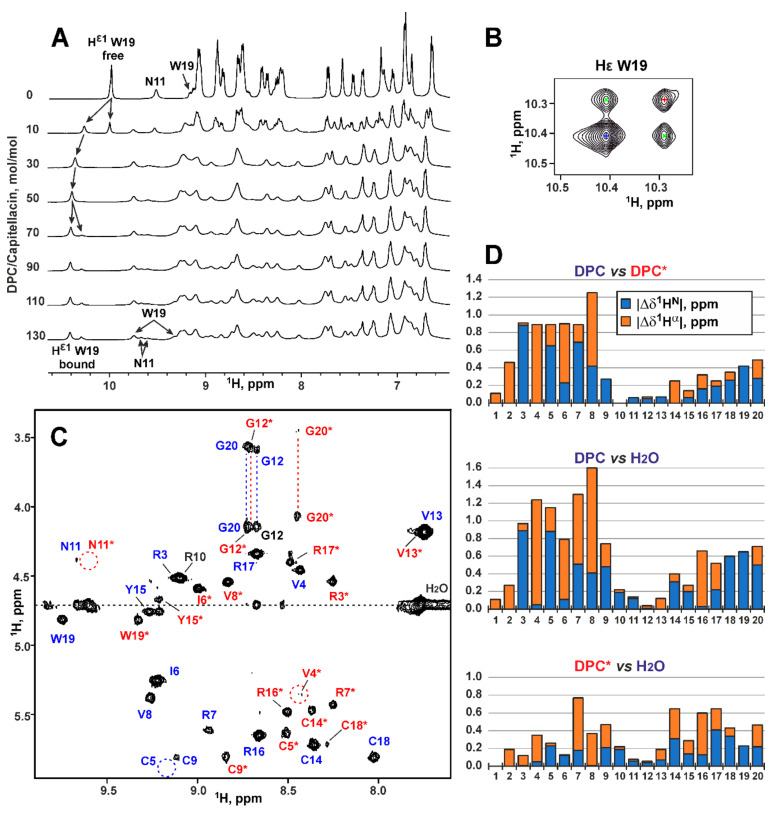
NMR data define the conformational heterogeneity of the micelle-bound capitellacin. (**A**). Titration of 0.5 mM capitellacin with DPC (5% D_2_O, pH 5.2, 30 °C). The HN-aromatic region of 1D ^1^H NMR spectrum at different DPC/capitellacin molar ratio is shown. (**B**). The fragment of 150 ms 2D ^1^H-NOESY spectrum showing exchange H^ε1^-H^ε1^ cross-peaks between the signals of Trp19 belonging to two structural forms of capitellacin in DPC micelles. The diagonal cross-peaks are marked by blue and red crosses. The exchange cross-peaks are marked by green crosses. (**C**). The fragment of the 2D ^1^H-TOCSY spectrum of capitellacin in DPC micelles (D:P = 130:1). The resonance assignment of H^N^-H^α^ cross-peaks is shown. The signals of the major and minor forms of the peptide are marked in blue and red, respectively. The signals of the minor form are additionally marked with asterisks. The signal common to both forms (Arg10) is marked in black. (**D**). Difference (absolute value) of ^1^H^N^ (blue) and ^1^H^α^ (orange) chemical shifts between the two forms of capitellacin in DPC micelles and major form of the peptide in water.

To confirm the absence of capitellacin dimerization in the membrane-mimicking environment we studied its structure by NMR spectroscopy in solution of dodecyl-phosphocholine (DPC) micelles. This environment was previously used for structural NMR studies of the arenicin-1 and arenicin-2 dimers [15,16,17]. Two sets of the capitellacin signals was observed upon addition of DPC to the capitellacin sample to the detergent-to-peptide molar ratio (D:P) of 10:1 (Figure 5A, two upper traces, H^ε1^ signal of Trp19). These sets corresponded to the peptide molecules being free in solution and bound to the micelles. Further increase in the DPC concentration to D:P of 30:1 led to disappearance of the free peptide signal, thus indicating complete micelle binding. At the same time, at D:P of 70:1 the second micelle-bound form of capitellacin became apparent in the spectra (Figure 5A). Further addition of DPC resulted in the narrowing of the signals of the second capitellacin form. To minimize an influence of this broadening, we used the D:P ratio of 130:1 for the structural study.

Analysis of integral intensities in the 1D ^1^H NMR spectrum and intensities of exchange cross-peaks observed in the 2D ^1^H-NOESY spectrum (Figure 5B) revealed that the relative population of two capitellacin forms was of 3:1 and the rate of exchange between them was of ~130 ms. The presence of exchange cross-peaks and relative broadening of the signals of the second peptide form significantly complicated the resonance assignment procedure. Nevertheless, the complete backbone resonance assignment and partial side-chain assignments were obtained for the both structural forms (Figure 5C). The examples of arenicin-1 and -2 showed that formation of the asymmetric peptide dimers should result in the doubling of backbone resonances. The presence of single resonance sets for both micelle-bound forms of capitellacin indicated that they corresponded either to the monomeric state of the peptide or to symmetric dimers. A thorough analysis of NOESY spectra did not reveal any NOE cross-peaks, which could support the presence of symmetric peptide dimers. At the same time, the observed NOE connectivity patterns revealed that both forms of capitellacin in micelles have a β-hairpin conformation similar to that observed in water. Thus, present data do not give any evidence of capitellacin dimerization in the membrane-mimicking environment.

The comparison of ^1^H^N^ and ^1^H^α^ chemical shifts (Figure 5D) revealed that two micelle-bound forms differed from each other by conformation of the *N*-terminal β-strand (Pro2-Val8). Thus, we suggest that these forms differ by the conformation of Ser1-Pro2 peptide bond or represent the peptide monomers with different degree of incorporation into the hydrophobic region of micelle (and this incorporation goes via the *N*-terminal β-strand). Interestingly, the chemical shifts of the second (minor) capitellacin form are much closer to the chemical shifts of the major peptide form in water (Figure 5D). Therefore, the minor form of the peptide in micelles either contains a *trans*-Ser1-Pro2 bond or corresponds to the peptide loosely associated with the micelle. At present, we cannot discriminate between these two possibilities. The low quality of NMR spectra in the micellar environment did not allow to analyze ^13^C chemical shifts for the Pro2 residue and study a topology of the peptide-micelle interaction. Development of special protocols for production of stable isotope (^13^C, ^15^N) labeled capitellacin variants are needed for the detailed structural study.

### 2.5. Proton Transfer Activity of Capitellacin

**Figure 6:**
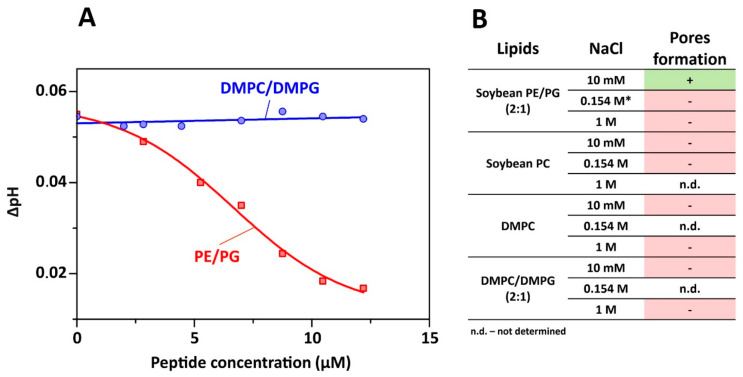
Proton transport measurements. (**A**) Dependence of the light-induced acidification of the bulk solution on the capitellacin concentration in the system with ESR proteoliposomes composed of DMPC/DMPG (2:1) or soybean PE/PG (2:1) lipids at 10 mM NaCl. (**B**) Comparative analysis of capitellacin pore-forming activity in different environment. * 5 μM tachyplesin-1 demonstrated proton transfer activity in this lipid system at 0.154 M NaCl.

Most amphipathic β-hairpin AMPs are known to penetrate bacterial membranes and at high concentrations induce pore-formation and/or cell lysis. To characterize the possible mechanism of capitellacin action, lipid-dependent pore formation was monitored by measurements of the proton transfer activity (PTA). Protonophore activity was analyzed using proteoliposomes containing a proton pump–bacteriorhodopsin from *Exiguobacterium sibiricum* (ESR). After incorporation into liposomes, ESR exhibits light-induced proton translocation, which causes a moderate decrease in the pH of the bulk solution corresponding to the proton pumping from the inside to the outside of liposomes [23]. The added peptide can dissipate the proton gradient across the liposomal membranes, increasing the pH value of the bulk solution to a certain level. Figure 6A shows the dependence of bulk pH values on the concentration of added capitellacin for two membrane systems. The absence of PTA in DMPC/DMPG (2:1) membranes and sigmoidal concentration dependence of ΔpH value in soybean PE/PG (2:1) liposomes were observed. This observation was rather unexpected, since both lipid systems contain the same proportion (~30%) of the anionic phosphatidylglycerol (PG) lipids. Thus, the observed difference in the PTA is primarily related to the difference in fluidity and cumulative curvature strain of the membranes. It is known that phosphatidylethanolamine (PE) lipids possess significant negative spontaneous curvature, while soybean lipids contain ≥ 50% unsaturated lipids (18:2 and 18:3) [24], which provide larger membrane fluidity. Both of these factors can enhance the peptide binding to the PE/PG bilayer surface as compared to the more rigid DMPC/DMPG membrane, which consists of the saturated lipids with almost zero spontaneous curvature.

Sigmoidal concentration dependence of PTA (Figure 6A) revealed cooperativity in the pore formation by capitellacin. The inflection point of the graph indicates the pore-forming concentration of ~6 μM. Activity of capitellacin in membranes of various lipid composition and at different salt concentrations is shown in Figure 6B. Interestingly, the proton transfer was observed only in the PE/PG lipid system and only at low ionic strength (10 mM NaCl). This indicates that electrostatic interactions of the positively charged peptide (overall charge +5) with the negatively charged component (PG) of the membrane are essential for the pore formation. In contrast, our experiments carried out under identical conditions revealed the pore formation by 5 μM tachyplesin-1 in soybean PE/PG liposomes at much larger (close to physiological) NaCl concentration (154 mM). Thus, two conditions are necessary for PTA manifestation by capitellacin: (1) the presence of negatively charged lipid (PG) and low salt concentration; (2) the presence of PE—the lipid with a negative spontaneous curvature. Although the interactions of AMPs with PE-containing bilayers was discussed in detail earlier [25,26], the dependence of the β-hairpin peptide activity on phosphatidylethanolamine lipids was discovered for the first time.

Bactericidal effect of the majority of AMPs is often considered to be due to their action on the lipid matrix with disruption of the bacterial cell membranes. Investigation of multiple AMPs by single bacteria cell imaging has allowed to detect local and stable pores at regions of high membrane curvature without uniform membrane coverage [27]. The PE/PG lipid system used in our study is considered as a good mimetic for the plasma membrane of Gram-negative bacteria. Thus, the pore formation observed in our study can be relevant also for perturbation of bacterial membranes. On the other hand, there are a variety of data indicating that β-structural AMPs can act via non−membranolytic mechanism. Indeed, in all our experiments liposomes were absolutely stable for many days after capitellacin collapsed the membrane potential. Previous studies of interaction of the β-hairpin AMP tachyplesin-1 and its analogs with lipids by solid-state NMR showed that membrane disruption is not correlated with antimicrobial activity of these peptides [28].

Bacteria maintain cytoplasmic pH which is compatible with optimal function and structural integrity of the cytoplasmic proteins supporting growth. Most non-extremophilic bacteria grow over the broad range of external pH values from 5.5 to 9.0 and maintain cytoplasmic pH within the narrow range of 7.4 ÷ 7.8. Hence, they are able to acidify or alkalinize the cytoplasm relative to the external medium. Acid and alkaline pH homeostasis have been extensively studied and reviewed [29]. Therefore, capitellacin can break the H+ circulation required for pH homeostasis and work of the Na^+^/H^+^ antiport system. At the same time, the strong dependence of proton transfer activity on ionic strength implies that the pore formation and dissipation of the pH gradient are not the main mechanism of the capitellacin antimicrobial action.

### 2.6. Biological Activity and Mechanism of Antibacterial Action

Antimicrobial activity of capitellacin, tachyplesin-1, and the reference antibiotic polymyxin B was assessed by broth microdilution assay determining minimum inhibitory concentrations (MICs) against a panel of clinically isolated and reference ATCC strains of Gram-negative and Gram-positive bacteria (Table 3). To minimize interactions of amphiphilic AMPs with polystyrene plate, serial dilutions of the peptides were performed in the presence of bovine serum albumin (BSA). To estimate therapeutic potential of tested peptides we used Mueller-Hinton medium with 0.9% NaCl, which might constrain an absorption of the peptides to the bacterial surface. 

**Figure 7:**
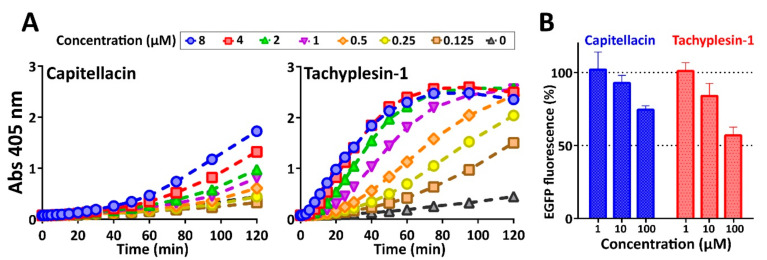
Comparative analysis of capitellacin and tachyplesin-1 antibacterial mechanism of action. (**A**) Kinetics of changes in *E. coli* ML-35p cytoplasmic membrane permeability at various peptide concentrations (from 0.125 to 8 µM, highlighted with colors) measured with the use of chromogenic marker—the product of ONPG (Abs 405 nm) hydrolysis. (**B**) Effects of the peptides on the fluorescence resulting from the in vitro translation of EGFP using *E. coli* BL21 (DE3) Star cell extract. The data are presented as the mean ± SD of three independent experiments.

Capitellacin exhibited high potency against most tested bacteria including XDR and metallo-β-lactamase producing strains which belong to ESKAPE pathogens; however, it was at least 2- to 8-fold lower than that of tachyplesin-1 (Table 3). This is not surprising, because tachyplesin-1 is known as one of the most active known AMPs, and its activity against Gram-negative bacteria is comparable to that for the last line antibiotic polymyxin B. Notably, all the peptides and polymyxin B were inactive against MDR *S. marcescens* at the tested concentrations—up to 32 µM. It is known, that *Serratia* spp. possess a natural (intrinsic) resistance to cationic AMPs because of the specific lipid composition of their membrane, which lacks an appropriate density of anionic binding sites [30]. The minimal difference in antimicrobial activity of the peptides against the panel of *E. coli* strains was shown with the median MIC values of 0.25 µM for capitellacin and 0.125 µM for tachyplesin-1. Next, the comparative analysis of kinetics of changes in *E. coli* cytoplasmic membrane permeability in the presence of the peptides was performed (Figure 7A).

Data on possible modes of tachyplesins action are quite controversial. The first proposed mechanism of tachyplesin-1 action on bacterial membrane suggested formation of anion-selective pores according to the “barrel-stave” model [31]. On the other hand, tachyplesin-1 does not aggregate and is too short for bilayer spanning and inducing transmembrane pores. Thus, tachyplesin-1-like AMPs were proposed to adopt the “in-plane diffusion” mechanism. In accordance with this model, AMPs are localized in parallel manner at the membrane surface and then exhibit significant segmental and global motions, causing membrane thinning and transient pores formation [32,33]. Our data confirm the pronounced membranolytic effect of tachyplesin-1 against *E. coli* at MIC and higher concentrations. Despite the longer hairpin length, capitellacin did not damage membrane at MIC. Moreover, even at the concentration of 32 × MIC (8 µM) a partial effect of cell membrane permeabilization was achieved after 2 h exposure. Similar effects were shown for some proline-rich antimicrobial peptide targeting bacterial 70S ribosome [34]. On the other hand, at higher concentrations some peptides like amyloid-beta (Aβ) can form oligomers and act via pore formation and subsequent membrane damage [35]. However, this finding contradicts the low amphiphilicity of the capitellacin structure and the absence of the peptide dimerization in model membranes even at the concentration of 3 mM that is far above the MIC value (Figure 4B and Figure 5C).

Considering the inefficiency of capitellacin to disrupt cytoplasmic membrane integrity at concentrations near the MIC value, we tested the ability of this peptide to inhibit protein biosynthesis in vitro (Figure 7B). The experiment was carried out using the bacterial cell-free protein synthesis system expressing the enhanced green fluorescent protein (EGFP). Streptomycin served as the positive control inhibiting EGFP synthesis at 1 µM that is in line with our previous data [34]. Both capitellacin and tachyplesin-1 only slightly inhibited this process at concentrations far above the MIC value (100 µM). This effect likely results from a non-specific interaction of cationic AMPs with nucleic acids that is known for a number of peptides [34,36]. The mechanism of capitellacin action does not seem to be related to the inhibition of bacterial translation therefore other molecular targets are possible. It seems that at concentrations near the MIC value, the peptide can cross the membrane without its rupture and interacts with some intracellular targets. Putative targets may be suggested when studying the mechanism of action of structurally similar β-hairpin AMPs arenicin-3 and tachyplesin-3. The first one was hypothesized to dysregulate the Gram-negative bacteria phospholipid transport pathways via the MlaC protein binding [37]. This pathway maintains lipid asymmetry in the outer membrane by retrograde trafficking of phospholipids from the outer membrane to the inner membrane. The tachyplesin-3 was found to bind both *E. coli* and *S. aureus* FabG, the conserved β-ketoacyl-acyl carrier protein reductase, thus targeting bacterial pathway for unsaturated fatty acid biosynthesis [38].

**Figure 8:**
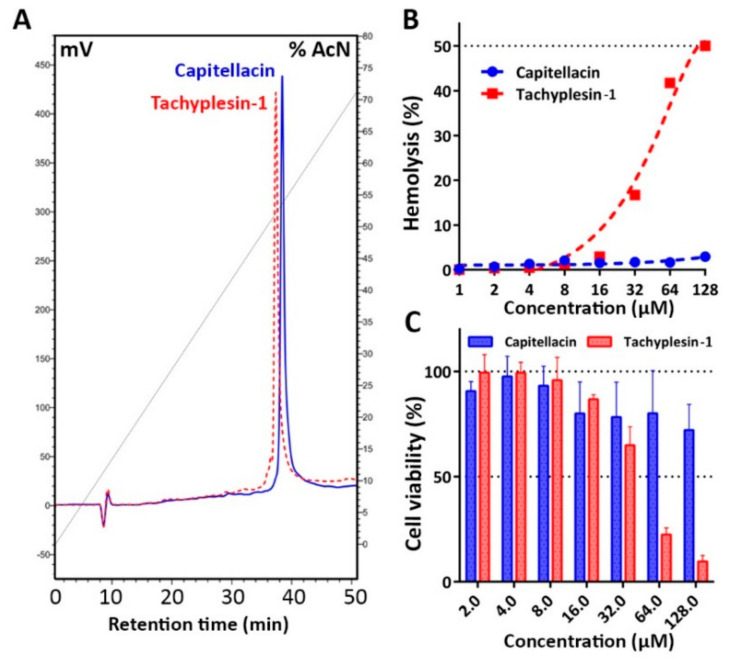
(**A**) Reversed-phase HPLC of recombinant capitellacin and tachyplesin-1 performed with a linear gradient of acetonitrile in water containing 0.1% TFA with the use of semi-preparative C18 column and detection at 214 nm. (**B**) Hemolytic activity of capitellacin and tachyplesin-1 after 1.5 h incubation (hemoglobin release assay). (**C**) Cytotoxicity of capitellacin and tachyplesin-1 against human embryonic fibroblasts (HEF) cell lines cells after 24 h incubation (3-(4,5-dimethylthiazol-2-yl)-2,5-diphenyltetrazolium bromide (MTT) dye reduction assay). The data are presented as the mean ± SD of at least three independent experiments.

Considering a low propensity of capitellacin to induce membrane disruption, next we analyzed its cytotoxic effects against adherent cell lines of human embryonic fibroblasts (HEF) as well as toward human red blood cells (hRBC). Tachyplesin-1 possesses a pronounced hemolytic activity with the HC_50_ value of 128 μM (Figure 8B). The obtained cytotoxicity data are in good agreement with those shown for hemolytic activity (Figure 8C). Tachyplesin-1 lysed 75% of the hRBC cells at the concentration of 64 μM. Noteworthy, capitellacin did not significantly affect both viability of fibroblasts and membrane integrity of erythrocytes at the concentration of 64 μM, which was 10–100-fold higher than antibacterial MICs measured *in vitro*. Interestingly, according to both calculated hydrophobicity index and experimental HPLC retention time, capitellacin is even more hydrophobic peptide as compared with tachyplesin-1 (Table 1, Figure 8A).

In the case of capitellacin, a low amphipathicity rather than hydrophobicity could be a key reason of the decreased cytotoxicity and overall membrane-active properties. One of the key structural differences between these peptides is the presence of Arg10 (capitellacin) and Tyr8 (tachyplesin-1) in the equivalent position of β-turn. We have previously shown that the replacement of Tyr8 by arginine in tachyplesin-1 minimizes cytotoxicity while decreasing antibacterial activity at least by 2–4-fold [39]. Interestingly, this analogue had a reduced potency to compromise *E. coli* ML-35p cytoplasmic membranes (unpublished data). A slight reduction of hydrophobicity by replacing Phe4 or Ile10 residues with alanine led to a sharp decrease in hemolytic activity as well [40]. The absence of key residues in the *N*- and *C*-terminal parts of the peptide, which are necessary for binding to lipopolysaccharide (LPS) as was shown for tachyplesin-1 [41], could be another reason of reduced activity of capitellacin against Gram-negative bacteria. Taking into account a low cytotoxicity of capitellacin, it is planned to improve its antimicrobial activity by designing a set of chimeric molecules that carry some structural elements of tachyplesin-1, in particular, the LPS-binding site.

## 3. Materials and Methods

### 3.1. Recombinant Production of the Peptides

Capitellacin and tachyplesin-1 were produced in a bacterial expression system as described previously [34]. Thioredoxin (Trx) was used as the fusion partner to ensure high yield of the peptide in the native conformation. The gene encoding capitellacin was obtained by annealing of two primers followed by one-round DNA-polymerase extension and then cloned into pET-based vector as described previously [39]. All the oligonucleotides used in this work were designed on the basis of *E. coli* K-12 codon usage bias. The target peptides were expressed in *E. coli* BL21 (DE3) as chimeric proteins that included 8×His tag, the *E. coli* thioredoxin A with the M37L substitution (TrxL), methionine residue, and a mature peptide. The cells transformed with the corresponding plasmid were grown at 37 °C in Lysogeny broth (LB) medium supplemented with 100 μg/mL ampicillin, 1 mM magnesium sulfate, 20 mM glucose, and were induced at OD_600_ 1.0 with 0.2 mM isopropyl β-D-1-thiogalactopyranoside (IPTG) for 5 h at 30 °C and 220 rpm. After centrifugation the pelleted cells were suspended and sonicated in the 100 mM phosphate buffer (pH 7.8) containing 20 mM imidazole and 6 M guanidine hydrochloride to fully solubilize the fusion protein. Purification of the peptide involved immobilized metal affinity chromatography (IMAC) of cell lysate with the use of Ni Sepharose (GE Healthcare), CNBr cleavage of the fusion protein, and reversed-phase HPLC (RP-HPLC) with the use of Reprosil-pur C18-AQ column (Dr. Maisch GmbH) as described in [34]. The collected fractions were analyzed by MALDI-TOF mass-spectrometry using Reflex III instrument (Bruker Daltonics). The fractions containing the target peptides were lyophilized and dissolved in water. The peptides concentrations were estimated using UV absorbance. The fractions with confirmed masses were dried in vacuo and repurified to estimate exact RP-HPLC retention times. RP-HPLC was performed using the same column at a flow rate of 2 mL/min in a linear gradient of solution B2 (80% acetonitrile, 0.1% TFA) in solution A2 (5% acetonitrile, 0.1% TFA): 0–100% for 70 min (Figure 4A).

### 3.2. NMR Spectroscopy and 3D Structure Calculation

NMR study was performed using a 1.5 mM sample of the recombinant capitellacin in 5% D_2_O at pH 5.2. The pH value of NMR sample was adjusted using concentrated HCl or NaOH solutions. For the NMR measurements in detergent micelles, d38-DPC (Anatrace) was added to the 0.5 mM peptide sample using aliquots of a concentrated water solution until the detergent-to-peptide molar ratio (D:P) of 130:1 was reached. The NMR spectra were measured at AVANCE 700 spectrometer equipped with room-temperature triple-resonance probe and at AVANCE-III 800 spectrometer equipped with cryoprobe (Bruker, Karlsruhe, Germany). The backbone and side chains resonance assignments were obtained by a standard approach using a combination of 2D ^1^H-TOCSY, ^1^H-NOESY, and ^13^C-Heteronuclear Single Quantum Coherence spectroscopy (HSQC) spectra in the CARA (version 1.84, Zurich, Switzerland) program. The ^3^J_H_^N^_H_^α^ coupling constants were determined from line shape analysis of NOESY and TOCSY cross peaks in the Mathematica program (version 8.0, Wolfram Research, Champaign, IL, USA). The ^3^J_H_^α^_H_^β^ coupling constants were estimated from the multiplet patterns in 2D TOCSY spectrum. The spatial structure calculations were performed in the CYANA (version 3.97) program [42]. Upper interproton distance constraints were derived from the intensities of NOESY (τ_m_ = 150 ms) cross-peaks via a “1/r^6^” calibration. Comparison of the intensities of the HN-HB2/3 cross-peaks showed that at this mixing time, the spin-diffusion effect does not strongly affect the signal intensities. Secondary structure of capitellacin was calculated from ^1^H and ^13^C chemical shifts using TALOS-N [43]. The φ and χ_1_ dihedral angles restraints and stereospecific assignments were obtained from J-couplings, NOE, and TALOS data. Hydrogen bonds were introduced using temperature gradients of amide protons (∆δ^1^H^N^/∆T), measured in the 20–45 °C temperature range in the 2D TOCSY and NOESY spectra. It was assumed that an amide proton with Δδ^1^H^N^/ΔT > −4.5 ppb/K could participate in the hydrogen bond formation. Additional upper/lower distance restraints were applied to restrain disulfide connectivity.

The secondary structure assignment was performed with STRIDE [44]. Visual analysis of the structures and figure drawings were performed using the MOLMOL program [45]. The disulfide conformation was described analogously to [46]. The χ^1^ and χ^1^′ angles of the disulfide were classified as usual: (−30°–−90°) − *g+*, (+30°–+90°) − *g-*, (−150° – +150°) − *t*, the χ^2^, χ^3^ and χ^2^′ angles were loosely classified as *p* for positive values and *n* for negative ones. The geometry of the β-hairpin peptides was analyzed as described in [14].

### 3.3. Accessing Codes

Experimental restraints, atomic coordinates, and chemical shifts of capitellacin in water solution have been deposited in PDB and BMRB databases under accession codes 7ALD and 34564, respectively.

### 3.4. Preparation of the Peptide-Containing Small Unilamellar Vesicles

The capitellacin (0.15–1.3 mg) was dissolved in methanol (0.6 mL) and mixed with required amounts of lipids in chloroform (0.6 mL) at 1:60 molar ratios. Then solvents were removed in rotary evaporator at 45 °C, and the samples were dried for 1 h under ~10^−3^ Torr. The peptide-lipid film was dissolved in 10 mM phosphate buffer (pH 7.2) to the final peptide concentration of 3 mM for FTIR, and 0.3 mM for CD measurements. The samples were incubated for 30 min at 20 °C and then were sonicated on ice for 1.5 min. Soybean phosphatidylethanolamine (PE), soybean phosphatidylglycerol (PG), and dimyristoylphosphatidylcholine (DMPC) were purchased from Sigma (St. Louis, MO, USA). Soybean phosphatidylcholine (PC) was purchased from Avanti Polar Lipids Inc. (Albaster, AL, USA). Dimyristoylphosphatidylglycerol (DMPG) was from Lipoid GmbH (Ludwigshafen, Germany).

### 3.5. Circular Dichroism Spectroscopy

Far-UV CD spectra were measured using a Jasco J-810 spectropolarimeter (Jasco, Tokyo, Japan) in demountable cells (Hellma Analytics, Müllheim/Baden, Germany) with 100 μm path length. Four scans were averaged.

### 3.6. Fourier-Transform Infrared Spectroscopy

FTIR spectra were measured on a Perkin-Elmer 1725 X Spectrometer (Perkin-Elmer, Beaconsfield, UK) with TGS detector and with hermetic interferometer area, which was sealed and fitted with two boxes of molecular sieves. The sieve boxes were baked at 250 °C for 8 h before measurements. Spectra in water and in aqueous suspension of liposomes were measured in very thin (12 μm) homemade demountable CaF_2_ cuvettes. 150 scans were averaged with a resolution of 4 cm^−1^.

### 3.7. Tryptophan Fluorescence and Quenching

Tryptophan fluorescence was measured by means of RF-5301PC spectrofluorophotometer (Shimadzu, Tokyo, Japan) fluorescence spectrophotometer using 1 × 0.4 cm quartz cuvettes (Hellma Analytics, Müllheim/Baden, Germany). Emission and excitation slits were 5 nm wide. The excitation wavelength was 280 nm. Fluorescence was quenched by addition of increasing amounts of 4 M potassium iodide.

### 3.8. Proton Transport Measurements

Protein expression, purification and spectroscopic characterization of the functional proton pump from *Exiguobacterium sibiricum* (ESR) was performed as described previously [23]. Reconstitution of ESR (from DDM) and formation of phospholipid proteoliposomes was carried out by cholate dialysis [47]. The protein to lipid molar ratio was 1:1700. 200 μL of proteoliposome suspension (protein concentration 0.25 mg/mL) was added to 2 mL salt solution so that lipid concentration in the cell was 2.5 mM. The peptide solution was added to the proteoliposomes with rapid stirring so that peptide to lipid molar ratio was in order of 1:500. The measurements were conducted in a thermostated cell at 25 °C with rapid stirring. Samples were illuminated with 500−Watt halogen lamp (OSRAM). pH was monitored with Cole-Parmer RZ-05658-65 electrode (Beverly, MA, USA) carefully shielded from radiation by foil.

### 3.9. Antimicrobial Assays

Gram-positive bacteria *Bacillus subtilis* B-886, *Micrococcus luteus* B-1314, *Staphylococcus aureus* 209P (ATCC 6538P) were obtained from All-Russian Collection of Microorganisms (Pushchino, Russia). The bacterial clinical isolates of Gram-negative bacteria (*Escherichia coli*, *Enterobacter cloacae*, *Acinetobacter baumanii*, *Klebsiella pneumoniae*, *Pseudomonas aeruginosa*, *Serratia marcescens*) were collected and provided by Solixant LLC (Solixant LLC, Moscow, Russia) and Sechenov First Moscow State Medical University hospital. The strains were characterized in our previous studies [17,18,19,20,21,22,23,24,25,26,27,28,29,30,31,32,33,34]. Other strains were obtained from American Type Culture Collection (ATCC, Manassas, VA, USA). Antimicrobial tests were performed as described previously [34]. Briefly, mid-log phase bacteria were diluted with the 2× Mueller-Hinton broth (MH, Sigma, St. Louis, MO, USA) supplemented with 1.8% NaCl or without it so that to reach a final cell concentration of 10^6^ CFU/mL. Fifty microliter aliquots of the obtained suspension were added to the same volume of the peptide solutions serially diluted with 0.1% water solution of bovine serum albumin (BSA) in 96-well flat-bottom polystyrene microplates (#0030730011, Eppendorf, Hamburg, Germany). After incubation for 24 h at 37 °C and 1000 rpm on the plate thermo-shaker (Biosan, Riga, Latvia), the minimum inhibitory concentrations (MICs) were calculated as the lowest concentration of peptide that prevented visible turbidity. To verify MIC values the respiratory activity of the bacteria was determined. Briefly, 20 µL of 0.1 mg/mL redox indicator resazurin (Sigma, St. Louis, MO, USA) was added to the wells, and the plate was incubated for an additional 2 h. The reduction of resazurin to resorufin was measured as the color change from blue to pink. The results were expressed as the median values of three experiments performed in duplicate. In all experiment series, no significant divergence was observed (within ±1 dilution step).

### 3.10. Bacterial Membranes Permeability Assay

The ability of the peptides to permeabilize the cytoplasmic bacterial membrane was accessed using a colorimetric assay with o-nitrophenyl-β-D-galactoside (ONPG, AppliChem, Darmstadt, Germany) and *E. coli* ML-35p strain constitutively expressing β-galactosidase. The final concentration of ONPG was of 2.5 mM. The concentration of the bacteria in each cell was of 2 × 10^7^ CFU/mL. Peptide samples were placed in a 96-well plate with a non-binding surface (NBS, Corning #3641, Corning, NY, USA), and the optical density of the solution was measured at 405 nm using the Multiskan EX microplate reader (Thermo Fisher Scientific, Waltham, MA, USA). The assay was performed in phosphate buffered saline (PBS) at 32 °C under stirring at 400 rpm. Control experiments were performed under the same conditions without the addition of peptide. Three independent experiments were performed, and the curve patterns were similar for all three series.

### 3.11. Hemolysis and Cytotoxicity Assay

The hemolytic activity of antimicrobial peptides was estimated against fresh human red blood cells (hRBC) using the hemoglobin release assay as described previously [39]. Four experiments were performed with hRBC from blood samples of independent donors. The colorimetric 3-(4,5-dimethylthiazol-2-yl)-2,5-diphenyltetrazolium bromide (MTT) dye reduction assay was used to determine the cytotoxicity of the peptides against human embryonic fibroblasts (HEF) cell line as described previously [48]. The experimental data were obtained from at least three independent experiments. The data are represented as average means ± standard deviations (SD).

### 3.12. Cell-Free Protein Expression Assay

In order to investigate effects of AMPs on the translation process, the peptides were added to a cell-free protein synthesis (CFPS) reaction mix with a plasmid encoding EGFP under the control of the T7 promoter. The *E. coli* BL21 Star (DE3) lysate required for the translation inhibition assay and reaction mixtures were prepared as described previously with some modifications [34]. In particular, the final concentration of plasmid DNA encoding EGFP was of 2 ng/μL. The peptides were dissolved in water with the addition of 0.05% BSA. The reaction volume was of 50 μL. Streptomycin was used as a positive control antibiotic. Fluorescence of the sample without peptide/antibiotic was set to 100%. The reaction proceeded for 60 min in a 96-well V-bottom black polypropylene microplates (#00306019043340, Eppendorf, Hamburg, Germany) in a plate shaker (30 °C, 1000 rpm). EGFP fluorescence (λ_Exc_ = 488 nm, λ_Em_ = 510 nm) was measured with a AF2200 microplate reader (Eppendorf, Hamburg, Germany). The experimental data were obtained from two independent experiments performed in triplicate.

## 4. Conclusions

This study extends the knowledge of the structure and biological functions of animal β-hairpin AMPs, in particular, of BRICHOS domain-related ones. In aqueous solution capitellacin exists as monomeric right-handed twisted β-hairpin and its structure does not reveal significant amphipathicity. Moreover, the peptide retains a monomeric conformation in membrane environments when incorporating into lipid bilayers. The obtained results suggest a potential medical application of capitellacin. The pronounced bactericidal activity against drug-resistant ESKAPE bacteria as well as a wider therapeutic window as compared with tachyplesin-1 makes capitellacin a promising broad-spectrum antibacterial agent. In contrast to other known β-hairpin AMPs, like arenicins, protegrins and tachyplesins, this peptide likely acts via non-membranolytic mechanism at concentrations inhibiting bacterial growth. An ability of capitellacin to compromise biological membranes was shown only at concentrations far above its MIC value measured in vitro against bacteria. As the translation inhibition was excluded, it is necessary to perform further in-depth study on searching possible intracellular targets of capitellacin, which can be identified by selection and investigation of resistant bacterial strains.

## Figures and Tables

**Table 1:** Characteristics of the recombinant AMPs.

Peptide	Recombinant Peptide Final Yield, mg/L	RP-HPLC Retention Time, min	Hydrophobicity Index ^1^/Hydrophobic Residues, % ^2^	Calculated [M + H]^+^ Monoisotopic Molecular Mass, Da ^3^	Measured Monoisotopic *m*/*z* Value ^4^
Capitellacin	6.1	38.5	−0.215/45	2379.16	2379.30
Tachyplesin-1	7.2	37.5	−0.482/47	2264.10	2263.73

^1^ Mean Kyte-Doolittle hydrophobicity index (GRAVY) calculated using the Expasy ProtParam tool. The maximum and minimum values of this index are +4.5 and –4.5 for poly-Ile and poly-Arg sequences, respectively. ^2^ Percentage of hydrophobic amino acids was calculated using the APD database. ^3^ Molecular masses were calculated by considering the presence of four Cys residues forming two disulfide bonds and ^4^ were determined experimentally using MALDI-TOF mass spectrometry.

**Table 2:** Capitellacin Trp fluorescence parameters in different environments.

Environment	Dielectric Constant	λmax (nm)	K_sv_ (M^−1^)
H_2_O	80.4	350	9.9
CH_3_OH	33.6	335	-
PE/PG	~2 *	332	3.1

* The dielectric constant ε = 2 characteristic for hydrocarbons is usually used for the hydrophobic part of the membrane.

**Table 3:** Antibacterial activity of capitellacin and known antimicrobial peptides.

Bacteria	Minimum Inhibitory Concentration (µM)		
	Capitellacin	Tachyplesin-1	Polymyxin B
**Gram-positive**			
*S. aureus* ATCC 6538P	8	1	n.d.
*M. luteus* B-1314	4	2	n.d.
*B. subtilis* B-886	16	2	n.d.
**Gram-negative**			
*E. coli* ATTC 25922	0.5	0.25	0.06
*E. coli* ML-35p	0.25	0.125	0.06
*E. coli* CI 214	0.125	0.06	0.03
*E. coli* (MDR CI 1057)	0.25	0.125	0.06
*E. coli* (MDR CI 3600)	0.5	0.06	0.06
*E. cloacae* (XDR CI 4172)	4	0.25	0.5
*A. baumanii* (XDR CI 2675)	0.25	0.016	0.5
*A. baumanii* (XDR CI 450)	0.5	0.06	0.03
*K. pneumonia* ATCC 700603)	4	1	0.125
*K. pneumonia* (MDR CI 358)	2	1	0.06
*K. pneumonia* (XDR CI 1056)	4	0.5	0.5
*K. pneumonia* (XDR CI 3395)	2	0.5	0.125
*P. aeruginosa* ATCC 27853	2	0.25	0.125
*P. aeruginosa* PAO1	2	0.25	0.25
*P. aeruginosa* (MDR CI 223 *)	8	1	0.5
*P. aeruginosa* (XDR CI 236 *)	4	0.5	0.5
*P. aeruginosa* (XDR CI 1049 *)	4	1	0.25
*P. aeruginosa* (XDR CI 1995 *)	8	1	0.125
*S. marcescens* (MDR CI 1689)	>32	>32	>32

n.d., not determined. CI, clinical isolate. MDR, multidrug resistant strain. XDR, extensively drug resistant strain. * metallo-beta-lactamase (MβL) producing strain.

## Supplementary Materials

The following are available online at https://www.mdpi.com/1660-3397/18/12/620/s1, Figure S1: MALDI-MS analysis of the recombinant capitellacin, Table S1: Statistics for the best CYANA structures of capitellacin.
