# Supplementary material for: Structure Elucidation and Functional Studies of a Novel β-hairpin Antimicrobial Peptide from the Marine Polychaeta Capitella teleta
Source: Mar Drugs. 2020 Dec 4;18(12):620. doi: 10.3390/md18120620 (PMC7761999; doi:10.3390/md18120620)
Supplement: Supplementary file 1 [file marinedrugs-18-00620-s001.pdf]

Supplementary material

Structure elucidation and functional studies of a novel β-hairpin antimicrobial peptide from the marine polychaeta *Capitella teleta*

Pavel V. Panteleev[1], Andrey V. Tsarev[1], Victoria N. Safronova[1], Olesya V. Reznikova[1], Ilia A. Bolosov[1], Sergey V. Sychev[1], Zakhar O. Shenkarev[1], and Tatiana V. Ovchinnikova[1,2]*

[1] M.M. Shemyakin & Yu.A. Ovchinnikov Institute of Bioorganic Chemistry, the Russian Academy of Sciences, Miklukho-Maklaya str., 16/10, 117997 Moscow, Russia; alarm14@gmail.com (P.V.P.); 79175492709@yandex.ru (A.V.T.); saf-victorianik@yandex.ru (V.N.S.); olesya-r@list.ru (O.V.R.); b_off2@mail.ru (I.A.B.); svs@ibch.ru (S.V.S.); zakhar-shenkarev@yandex.ru (Z.O.S.); ovch@ibch.ru (T.V.O.)

[2] Department of Biotechnology, I.M. Sechenov First Moscow State Medical University, Trubetskaya str., 8-2, Moscow, 119991, Russia; ovch@ibch.ru (T.V.O.)

*Correspondence: ovch@ibch.ru; Tel.: +7-495-336-44-44

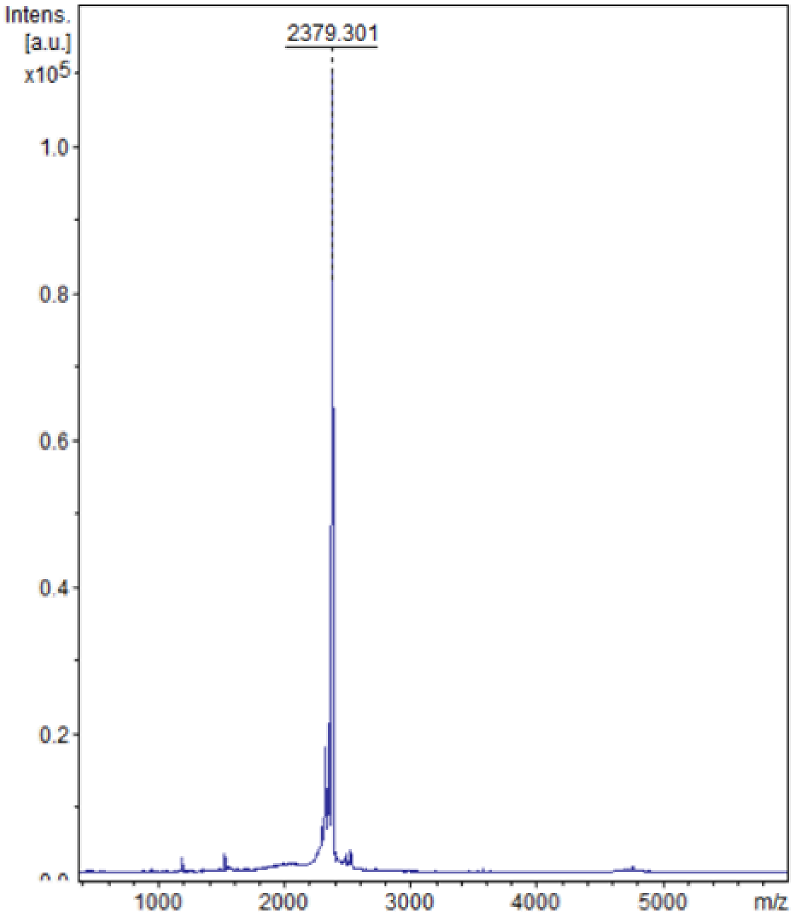

Figure S1. MALDI-MS analysis of the recombinant capitellacin. The experimental $[M+H]^+$ monoisotopic mass is indicated in the picture.

Table S1. Statistics for the best CYANA structures of capitellacin.

**Distance and angle restraints**	
Total NOE contacts	157
intraresidual	70
sequential (|i-j|=1)	53
medium-range (1<|i-j|<4)	6
long-range (|i-j|>4)	28
Hydrogen bonds restraints (6 bonds, upper/lower)	12/12
S-S bond restraints (2 bonds, upper/lower)	6/6
Torsion angle restraints	25
Angle φ	16
Angle $\chi 1$	9
**Total restraints/per residue:**	218/11
**Statistics for calculated structures**	
Structures calculated/selected	200/20
CYANA target function (Å^2)	0.22±0.3
Violations of restraints	
Distance (>0.2 Å)	2
Distance (>0.4 Å)	0
Dihedral angles (>1°)	0
**RMSD (Å) overall (Ser1-Gly20)**	
Backbone	0.42±0.15
Heavy atoms	1.13±0.13
**Ramachandran analysis (backbone)**	
Residues in favored regions (%)	75%
Residues in allowed regions (%)	25%
Residues in disallowed regions (%)	0%
